# Emerging Self-Representation Presents a Challenge When Perspectives Conflict

**DOI:** 10.1162/opmi_a_00065

**Published:** 2022-11-22

**Authors:** Emanuela Yeung, Dimitrios Askitis, Velisar Manea, Victoria Southgate

**Affiliations:** Department of Psychology, University of Copenhagen

**Keywords:** infancy, pupillometry, perspective tracking, self, inhibition

## Abstract

The capacity to take another’s perspective appears to be present from early in life, with young infants ostensibly able to predict others’ behaviour even when the self and other perspective are at odds. Yet, infants’ abilities are difficult to reconcile with the well-known problems that older children have with ignoring their own perspective. Here we show that it is the development of the self-perspective, at around 18 months, that creates a perspective conflict between self and other during a non-verbal perspective-tracking scenario. Using mirror self-recognition as a measure of self-awareness and pupil dilation to index conflict processing, our results show that mirror recognisers perceive greater conflict during action anticipation, specifically in a high inhibitory demand condition, in which conflict between self and other should be particularly salient.

## INTRODUCTION

The capacity to take another’s perspective appears to be present from early in life, with young infants are ostensibly able to predict others’ behaviour even when the self and other perspective are at odds (Baillargeon et al., 2010; Choi et al., 2018; Luo & Johnson, 2009; Southgate & Vernetti, 2014). Yet, infants’ abilities are difficult to reconcile with the well-known problems that 3-year-olds have with ignoring their own perspective (Birch & Bloom, 2003). A particular challenge for explaining the processes that underlie infants’ abilities is to account for the role that developmentally-dependent abilities like conflict monitoring and inhibitory control seem to play in managing perspective conflict in older children (Carlson & Moses, 2001; Kloo & Perner, 2003).

The false belief task is the paradigmatic case of perspective conflict because, to make the correct inference, one cannot rely on one’s own (differing) representation and many studies have demonstrated some involvement of inhibitory resources in success on verbal mentalizing tasks (Devine & Hughes, 2014). This relationship likely stems from the assumed need to deal with two conflicting representations: one derived from the child’s own knowledge of reality and the other from the child’s encoding of the other’s perspective. To resolve this conflict, various accounts appeal to conflict monitoring and management mechanisms (Leslie et al., 2004). Neuroimaging work supports this view by demonstrating that brain regions implicated in inhibition are required for success on perspective conflict tasks (Hartwright et al., 2015; Samson et al., 2005) and recruited at a point where the participant needs to make their judgement (McCleery et al., 2011). Given that young infant’s inhibitory skills are likely to be less developed than those of preschoolers (Hendry et al., 2016; Müller & Kerns, 2015), it remains unexplained how infants as young as 6 months of age seem to make predictions about another’s behaviour when the infant and the other should have conflicting representations (Luo & Baillargeon, 2007; Southgate & Vernetti, 2014).

One possible explanation could be that the non-verbal measures with which infants’ abilities are measured do not place demands on inhibition, and thus enable infants to reveal their perspective tracking ability by removing the need to inhibit their own perspective (Baillargeon et al., 2010). While this would be a neat solution, it is unclear why non-verbal tasks should not demand conflict resolution, especially in the scenarios described above (e.g., Luo & Baillargeon, 2007; Southgate & Vernetti, 2014) where the infant is still faced with two conflicting perspectives and should need to ignore their own perspective to make the correct prediction. It seems unlikely that the nonverbal nature of the task circumvents the need for inhibition since dual-task manipulations, targeting executive functions, impair adults’ performance on an analogous nonverbal perspective conflict task (Schneider et al., 2012). Furthermore, manipulating the presence or absence of the object, which targets inhibitory demands in adults (Hartwright et al. 2015; Samson et al., 2005), impairs both children and adults’ ability to make belief-based action predictions on a nonverbal task (Wang & Leslie, 2016).

Recently, an alternative account has instead argued that young infants’ precocious success on tasks involving perspective conflict may be due to an altercentric – or other-centred - bias in infant cognition (Southgate, 2020). According to this hypothesis, an altercentric bias emerges from two features of early cognition. First, like adults, infants experience interference from spontaneous encoding of the other’s perspective. For example, 7-month-old infants look longer to the absence of a ball when another agent mistakenly believes it should be present, even if the infant should know that it is absent, than they do when both they and the agent both believe it to be absent (Kovács et al., 2010). Together with data from adults showing modulation by the others’ perspective (e.g., Kovács et al., 2010; Samson et al., 2010), this suggests that both infants and adults spontaneously encode events from the perspective of other agents who are present. Second, an initial absence of a competing self-perspective in the first year of life reduces the potential for perspective conflict (when perspectives conflict), allowing the altercentric encoding to be the dominant influence on infants’ memory. This hypothesis draws on evidence for a late emergence of cognitive self-representation between 18 and 24 months of age and proposes that self-awareness is a prerequisite for generating a self-perspective. It also draws on the self-reference effect (Rogers, 1977; Symons & Johnson, 1997) and hypothesizes that only when the child is able to generate a representation of their own perspective (e.g., the ball is in the left-hand box) does this representation become a competitor to the representation of the object that is highlighted or cued by another agent’s attention (the ball is in the right-hand box). Thus, in the absence of the self-perspective, young infants are proposed to have an altercentric bias that tracks the perspective (i.e., follows the attention) of another individual, allowing infants to generate representations of events and make predictions about future events, without the need to inhibit or control the conflicting self-perspective (Southgate, 2020).

While there is already evidence that infant’s encoding and memory of events can be enhanced and changed by others’ attention (e.g., Reid & Striano, 2005; Yoon et al., 2008), recent evidence suggests that when a conflict in perspectives exists, 8-month-old infants better remember the event that was witnessed by the other agent, than they do the event witnessed alone, evidencing a memory error for the object’s location that was co-witnessed with another agent (Manea et al., 2022). Although these infants are well below the age at which self-representation appears to emerge, and thus the data are consistent with the hypothesis that an absence of self-representation reduces the experience of perspective conflict leading to a preferential encoding of events from the other’s perspective, it does not address the role of emerging self-representation. In the present preregistered study, we tested the hypothesis that the development of self-awareness between 18 and 24 months is instrumental in creating a perspective conflict, and challenge, for young children. Based on the hypothesis that conflict in a false-belief scenario arises because of a mismatch between the self-perspective and other-perspective, we investigated whether experienced conflict was greater in infants who have achieved self-awareness, a presumed prerequisite for awareness of the self-perspective. Our main question was whether a manipulation aimed at increasing or decreasing the demands on participants’ inhibitory resources in a perspective-conflict scenario, would a) be detected only by infants who exhibit evidence of self-awareness and b) modulate belief-based action predictions only in infants who exhibit evidence of self-awareness.

In line with previous work (Hartwright et al., 2015; Samson et al., 2005; Wang & Leslie, 2016), we manipulated demand on inhibitory resources by either transferring an object from one box to another (high-conflict, HDfb) or removing the object from the scene altogether (low-conflict, LDfb). In both cases, there is a conflict in perspectives between the child and the agent, but that conflict is thought to be greater when the object remains in the scene as a salient reminder of the participant’s own knowledge. We used the mirror self-recognition (MSR) test as an index of self-awareness, categorizing infants as either recognisers or non-recognisers. While MSR is a direct measure of physical self-recognition, there is considerable evidence that it also indexes so-called ‘objective’ self-awareness, relating to self-other comparison (Kampis et al., 2022), dyadic imitation (Asendorpf & Baudonnière, 1993), personal pronoun use (Lewis & Ramsay, 2004), and brain indices of self-related processing (Bulgarelli et al., 2019).

We preregistered the prediction that recognisers would perceive greater conflict in the high- than low-conflict scenario, whereas the perceived conflict between the two conditions would be less for non-recognisers. To measure this, we used pupil size as an index of perceived conflict (Rondeel et al., 2015; Sirois & Brisson, 2014), predicting greater pupil dilation in the high than low perspective conflict events, especially in infants who evidence mirror self-recognition. Change in pupil diameter has been used as a marker of cognitive effort in both adults and infants (Hess & Polt, 1960; Kahneman & Beatty, 1966; Kaldy & Blaser, 2020) and pupillometry studies with young infants have shown that infants’ pupils dilate in response to violations of expectation or prediction (Gredebäck & Melinder, 2010; Jackson & Sirois, 2009; Pätzold & Liszkowski, 2019; Sirois & Jackson, 2012; Zhang & Emberson, 2020; Zhang et al., 2019).

Secondly, we asked whether self-awareness would modulate belief-based action prediction, reasoning that experiencing conflict between perspectives would impair children’s action prediction capabilities in the absence of sufficient inhibitory resources. Thus, we preregistered the prediction that infants who had achieved mirror self-recognition would show a greater tendency towards incorrect action anticipation than those who had not. We further aimed to test the prediction that there would be a relationship between inhibitory control and anticipatory looking on the HDfb condition, but only in those infants who had achieved self-awareness. In other words, we reasoned that for those infants who did experience the conflict in perspectives, better inhibitory control ability would allow them to prioritise the other’s perspective and make the correct anticipation.

## METHOD

This study was preregistered on the Open Science Framework and the description of the testing protocol, stimuli, and planned analyses can be found here: https://osf.io/thr7y.

### Participants

To reach our pre-registered sample of 50 infants (*n* = 25 in each group of mirror recognisers and mirror non-recognisers) we tested 80 infants in total. Participants were included based on providing sufficient eye-tracking data for analysis (described below) and completing the Mirror Self-Recognition (MSR) task. Of the 50 infants included in the non-verbal perspective-tracking task (*M*_age_ = 18.17 months, *SD* = 9.83 days, 20 females), *n* = 40 also successfully completed the inhibition task. There was no significant age difference between mirror recognisers and non-recognisers (*M*_recognisers_ = 18.22 months ± 7.26 days, *M*_non-recognisers_ = 18.12 months ± 11.83 days, *t*(39.84) = −1.10, *p* = .28, Welch’s test).

The study was approved by the Research Ethics Committee of the Faculty of Social Sciences at the University of Copenhagen and parents provided informed consent prior to participation.

### Procedure

Infants were most often accompanied by one parent, who was asked to maintain a neutral presence throughout the session. The task order for all participants was the same: the non-verbal perspective-tracking task (NVPT), the Early Childhood Inhibitory Touchscreen Task (ECITT), and the Mirror Self-Recognition task (MSR).

### Non-Verbal Perspective-Tracking Task

#### Apparatus.

Gaze and pupil diameter data were recorded from both eyes at 500 Hz using an Eyelink1000 Plus eye tracker (SR Research, Ontario, Canada). The stimuli were presented on a 17″ monitor.

#### Stimuli.

Animations were created using Blender (Blender Online Community, 2018) and Adobe Premiere Pro. We ensured that our stimuli were isoluminant across the duration of each trial and between conditions, to minimize pupil changes caused by variations in luminosity.

#### Design and Procedure.

Infants were seated in their parent’s lap, approximately 60cm from the screen and Eyelink’s 5-point calibration was used for each infant. Stimuli were presented using MATLAB for Mac (R2016b) and Psychtoolbox (3.0.16 Beta, SVN revision 10420).

**Figure 1.**
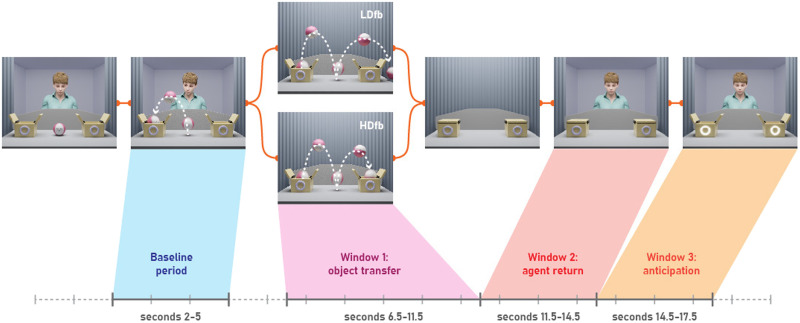
Time course and structure of the Non-Verbal Perspective-Tracking Task.

The sequence of events is shown in Figure 1. Infants were first presented with two familiarization trials. On each trial, an agent observed a ball jumping into one of two boxes. Next, two lights illuminated together with a sound cue, and the agent reached into the box to retrieve the ball. Infants saw the agent reaching once into the left box and once into the right box with order counterbalanced.

Following familiarization, test trials were presented in blocks, each consisting of 11 trials. On each test trial, the agent witnessed a ball move into one of two boxes. A curtain was then drawn shut, occluding the agent, and the ball then jumped into the other box (false belief – high demand condition, HDfb, movie 1) or out of the scene (false belief – low demand condition, LDfb, movie 2). The curtains reopened, revealing the agent, the boxes reopened, and the lights on each box illuminated together with a sound cue^1^. In each block, participants saw 5 videos for each false belief condition, including 2 trials where the agent reached into the box where they last saw the ball (the “outcome”) after the anticipation phase. In addition, infants also saw 1 “true belief” trial in each block in which the agent witnessed the ball moving into the second box and then reached into the box where the ball was located. The purpose of the “outcome” and true belief trials was to motivate infants to make anticipatory looks throughout the block (see below), but the gaze and pupil data from these trials were not used for analysis.

Infants were excluded if they looked away during portions of the familiarization and/or test trials that were critical for understanding the structure of the task (see supplementary information for further details).

#### Pupil Diameter.

The eye-tracking data was extracted from the eye-tracker using the Eyelink IDE and custom MATLAB scripts. The raw pupil data was first converted from arbitrary units to millimeters. Next, we examined the data obtained from each eye for each participant and excluded data from one eye when the ratio of the pupil standard deviations between eyes was less than 1.5. The subsequent steps of our pre-processing pipeline followed the procedure described by Kret and Sjak-Shie (2019); for details, see the supplementary information. Due to preprocessing, we removed 20.7% (*SD* = 15.4%) of pupil data on average for each participant. We also removed trials with more than 30% of data missing, which resulted in acceptance of 835 out of 1086 trials. Our final sample consisted of infants who provided at least 3 trials per condition after pre-processing, resulting in 707 trials included in total.

Our primary dependent measure was change in pupil dilation. We calculated the change in pupil dilation by subtracting the pupil size at each timepoint from a baseline period. We defined the baseline as the average pupil diameter between 2000–5000 ms, which corresponds to the movement of the ball from the centre of the screen into the first box and is identical in both conditions. In order to ensure that pupil data was not contaminated by noise in the baseline, we restricted the analysis of pupil data to trials that had no missing values in their baseline. This resulted in a further exclusion of 51 trials, leaving 656 trials in total.

The test phase was the period between 6.5 to 17.5 s of each trial. Previous studies have found that infants’ pupils react more slowly than those of adults’ and require more time to adapt to changes in stimuli (Pätzold & Liszkowski, 2019; Verschoor et al., 2015). As such, the time windows for analyses of change in pupil size were adjusted to 500 ms after event boundaries. Because we did not have a priori information about when infants might experience a conflict between the self- and other-perspective, we pre-registered three possible windows of analysis: 1) 6.5–11.5 s, when the ball moved from the first to the second box, 2) 11.5–14.5 s, when the agent returned and the boxes opened, and 3) 14.5–17.5 s, when the sound and light cued for action prediction and while the agent sat stationary behind the boxes (see Figure 1 for an overview of our task design).

#### Gaze and Anticipatory Looking.

Fixations were computed online by the Eyelink system using the cognitive configuration (see Eyelink manual 2017, 4.3.9), and fixations from both eyes were used when available to assess fixation on areas of interest (AOIs) or on the screen.

We preregistered two dependent measures to analyse our anticipatory looking data. Our first dependent measure, “first fixations”, was binary: it expressed whether the first box AOI that the participant fixated on within the time window of interest was the correct side. We defined an AOI that included the two boxes (see supplementary information). The time window of interest was initially defined as the 3000 ms interval starting from the sound and light cue onset. However upon reviewing the gaze data and video recordings from our participants, we found that many infants already made anticipatory saccades when the boxes reopened, 835 ms prior to the light cues on the boxes. Thus, we adjusted our time window of interest to begin when the boxes opened. First fixation scores were averaged across all trials per condition for each participant.

The second dependent variable was a differential looking score (DLS) calculated for each participant as cumulative fixation time to correct box AOI divided by the sum of cumulative fixation times to the two box AOIs. The cumulative fixation times were calculated within 3000 ms from the box opening cue onset. DLS scores were averaged across all trials per condition for each participant. In addition to the exclusion criteria described above, we also excluded trials where the child did not make fixations in either box AOI within the 3000 ms from cue onset.

### Early Childhood Inhibition Touch Screen Task

The Early Childhood Inhibitory Touchscreen Task (ECITT; (Holmboe et al., 2021) is a computerized touchscreen task designed to assess inhibitory control in infants and toddlers (see supplementary information for additional details regarding the materials and procedure).

### Mirror Self-Recognition Task

#### Procedure.

We conducted a mirror self-recognition task (following the procedure of Bulgarelli et al., 2019; see also Amsterdam, 1972) to assess children’s ability to recognize themselves in the mirror. The testing procedure included four phases: 1) children were first exposed to and familiarized with the mirror prior to application of the mark, 2) the mark was applied to the child’s nose surreptitiously after mirror was occluded, 3) the child was exposed to the mirror again after the mark was on their nose, and 4) the experimenter pointed to the child’s reflection in the mirror and asked “Who is that?”.

#### Coding.

Children’s reactions in the mirror were coded by a trained research assistant with respect to whether they touched the mark on their nose during phase 3 (mark-directed behaviour) and whether they verbalized any self-reference when seeing themselves in the mirror during phase 3 or upon request during phase 4. Children were considered to have passed the mirror test (“mirror recognisers”) if they showed mark-directed behaviour, used a first-person pronoun, or used their own name. Children who did not exhibit these behaviours were considered “mirror non-recognisers”. None of the children touched the mark during the second phase before seeing themselves in the mirror. A subset of participants (50%) were double-coded by a second independent coder. Coders agreed in 86% of all cases (κ = .72). In cases of disagreement, a third coder was involved, and the final score was decided by the majority. All coders were blind to children’s performance on the NVPT and ECITT.

## RESULTS

### Pupil Dilation

After pre-processing, infants in our sample watched a mean of 5.46 ± 2.05 HDfb and 5.44 ± 1.82 LDfb trials. There was no significant difference in the number of trials included per condition (*t*(49) = 0.10, *p* = 0.92). When split by MSR status, there were also no differences in number of trials included in both the HDfb (*M*_recognisers_ = 5.00 ± 1.98, *M*_non-recognisers_ = 5.92 ± 2.06, *t*(48) = 1.61, *p* = 0.11), and LDfb conditions (*M*_recognisers_ = 5.04 ± 1.65, *M*_non-recognisers_ = 5.84 ± 1.93, *t*(48) = 1.58, *p* = .12).

**Figure 2.**
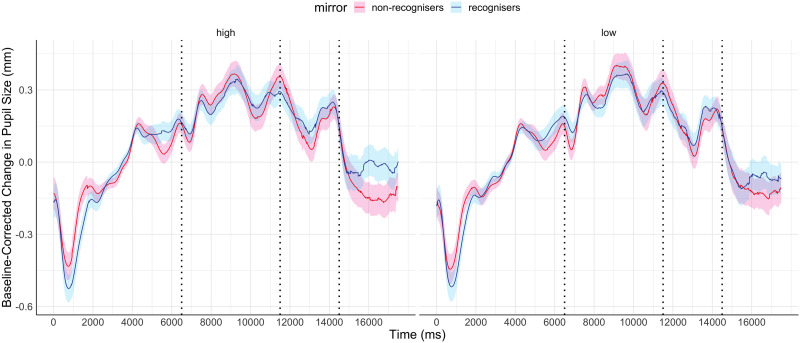
**Baseline-corrected change in pupil size across the duration of the trial.** The panel on the left shows the HDfb condition, the panel on the right shows the LDfb condition. Shaded regions show 95% confidence intervals. The dashed vertical lines demarcate the beginning of each pre-registered time window.

Figure 2 shows the mean baseline-corrected pupil diameter for the duration of the trial for both groups of participants across the two conditions. To analyse differences in pupil dilation, we pre-registered analyses using both ANOVA and functional data analysis (FDA). One participant (a mirror recogniser) did not have sufficient data in window 3 and was excluded from the ANOVA for this time window and the FDA analysis.

First we looked at differences in dilation using a 2 (condition) × 2 (MSR status) repeated-measures ANOVA, with pupil dilation averaged within each of the 3 pre-registered time windows. However, there were no significant main effects or interactions in any of the time windows (see supplementary information for details). Although averaging across time windows or trials is common practice for other measures of gaze data, this approach may not be optimal for pupil data due to loss of information (Jackson & Sirois, 2009). For this reason, we also pre-registered FDA (Ramsay & Silverman, 2002) to assess whether the change in pupil dilation over time differed between our two groups and to determine the specific time windows in which differences could be observed.

The data was fitted using B-spline functions of order 4 with 58 bases. We performed a functional two by two mixed ANOVA (Ramsay & Silverman, 2002) with mirror status as the between groups factor and demand condition (HDfb and LDfb) as the repeated measure. To determine statistical significance thresholds, we performed permutation analysis at 0.05 significance level and 10000 permutations, where the mirror and demand conditions were randomly shuffled. For each permutation, the computed F-statistics were used to form null distributions, against which our original values were compared. When we examined mirror status (the between groups factor), we detected a significant difference in pupil dilation between mirror groups in the anticipation window between 15918–16384 ms and 16544–16904 ms. When we compared dilation between conditions, there was a statistical difference in the time period that differed visually between the conditions (i.e., when the ball jumps out of the scene or into the second box). We did not find any other statistical differences in pupil dilation between the demand conditions, nor in their interaction with the mirror factor (see Figures 3a–3c). When looking only in the HDfb condition, a functional *t*-test also showed that baseline-corrected pupil dilation differed between groups in the anticipation phase, specifically between 15790–16990 milliseconds. No differences between pupil dilation were detected in the LDfb condition (see Figures 4a–4b).

**Figure 3a.**
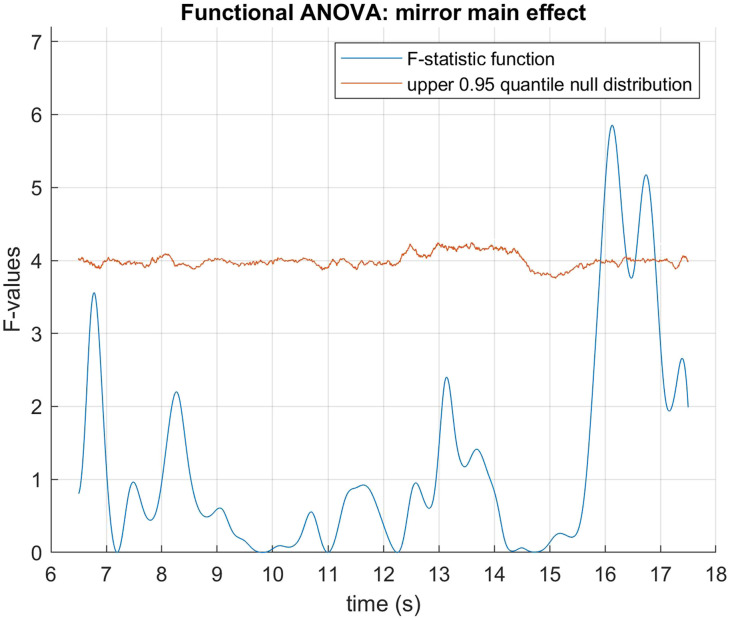
**Functional F-test (ANOVA) with MSR status as the between-subjects factor.** Statistical significance was detected during the outcome phase, between 15918–16384 and 16544–16904 milliseconds.

**Figure 3b.**
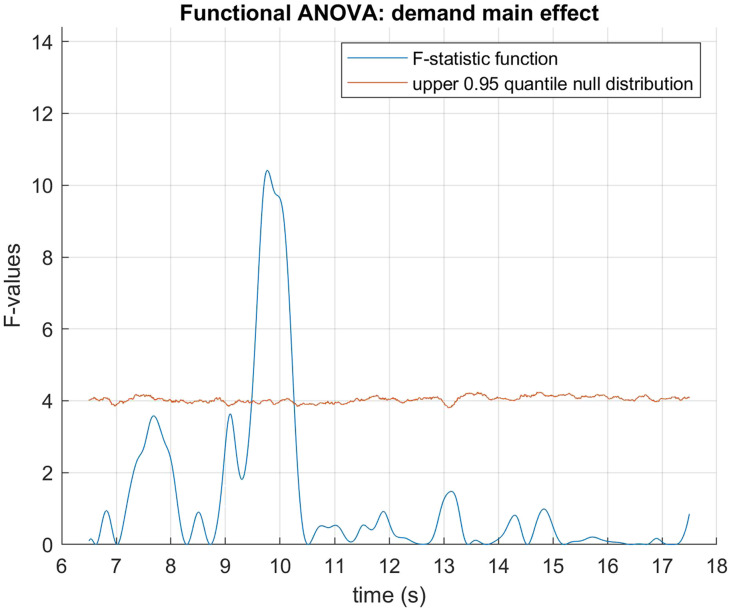
**Functional F-test (ANOVA) with condition (HDfb vs. LDfb) as the between-subjects factor.** Statistical significance was detected only around the time period when the two conditions differed visually (i.e., the ball jumping into the second box or jumping out of the screen).

**Figure 3c.**
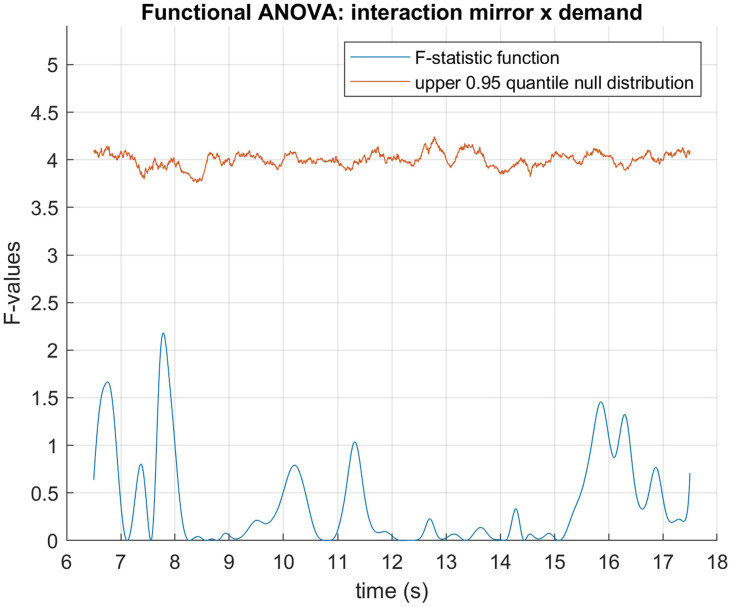
**Functional F-test (ANOVA) for the interaction between MSR status and demand condition.** No statistical significance was detected.

**Figure 4a.**
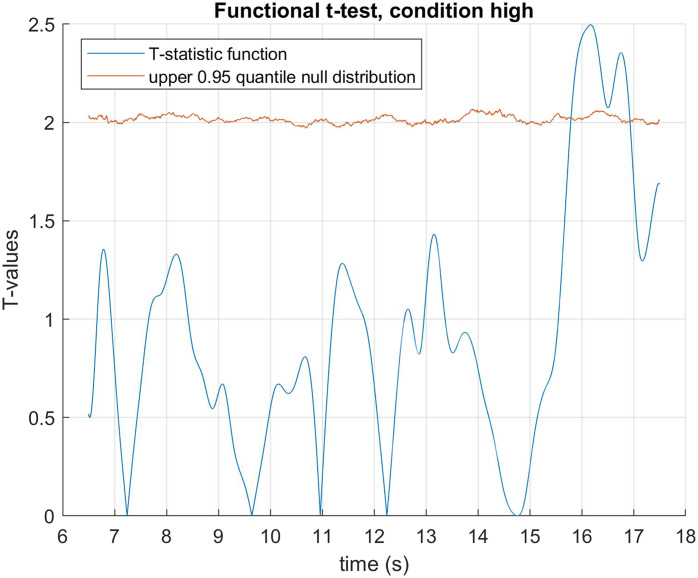
**Functional *t*-test for mirror recognisers vs non-recognisers within the high demand condition.** Statistically significant differences were observed during the outcome phase, from 15790 to 16990 milliseconds.

**Figure 4b.**
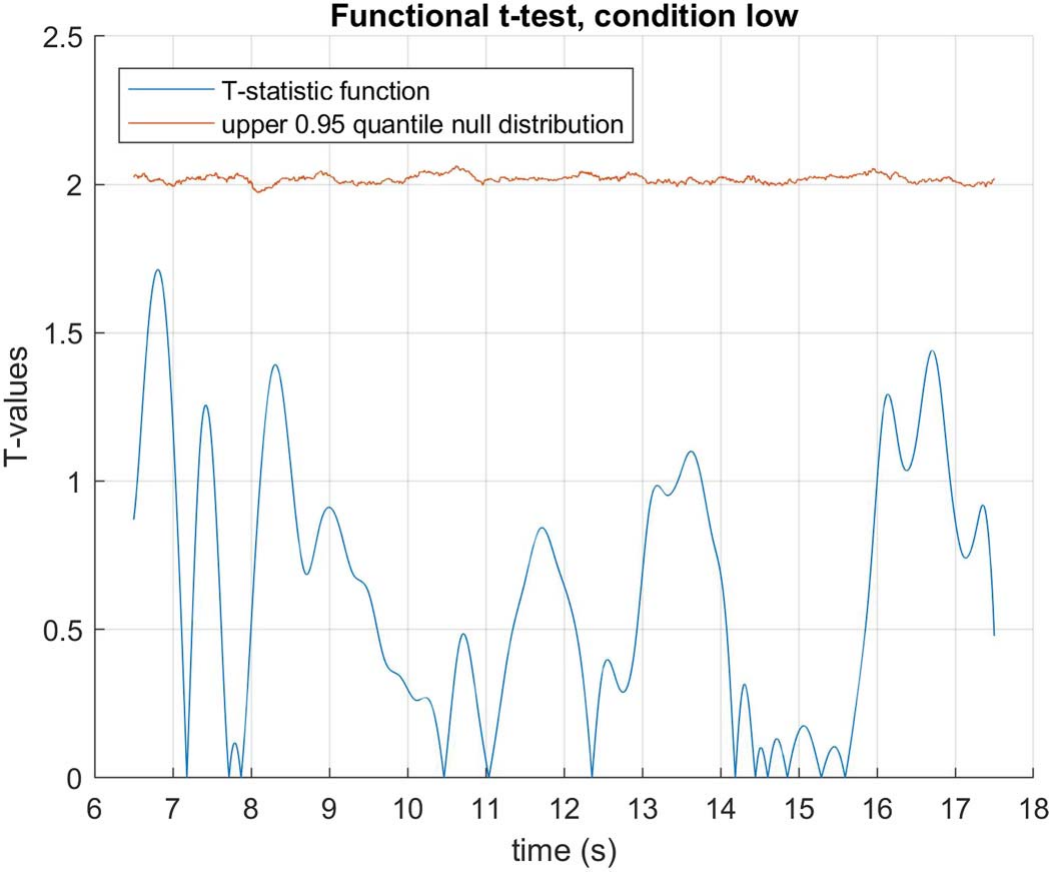
**Functional *t*-test for mirror recognisers vs. non-recognisers within the low demand condition.** No statistically significant differences were observed across the test phase.

#### Exploratory Analyses

##### Growth Curve Analysis.

To provide further support for the effect of MSR status detected in the third pre-registered time window (14.5 s–17.5 s) on pupil dilation, we performed a post hoc growth curve analysis (GCA) to examine how the pupil reactions deviated over time. GCA is a multilevel regression model that fits orthogonal polynomials to time series data to model variations in the curve shape (Mirman, 2017). It has been increasingly used in modelling pupil data time series (Kuchinsky et al., 2013; McGarrigle et al., 2017; Wagner et al., 2019; Winn, 2016) and is ideal for investigating the temporal dynamics of pupil data that can be obscured in a time window averaging approach.

We used normalised orthogonal polynomial terms up to degree 4 to model the pupil data from 14.5 s to 17.5 s (see supplementary information for further details). Given that our FDA analysis revealed effects only in the HDfb condition, we restricted our analysis in this condition. We considered the full model with mirror status as a main effect and all its interactions with polynomial terms (see Table 1). Based on a Likelihood Ratio Test, the full model fit the data better than the null model (i.e., the one consisting of the polynomial predictors but without the mirror factor; χ^2^ = 11.521, dDF = 5, *p* < 0.05). Following Mirman (2017), we examined each individual interaction by dropping it from the full model and evaluated its unique effect by the reduction in the model fit.

**Table 1.** The full model within the high demand condition, with pupil ∼ mirror * (op1 + op2 + op3 + op4) + (op1 + op2 + op3 + op4|subj). For the calculation of the *p*-values the *z*-distribution was used as an approximation to the t-distribution (see Mirman, 2017). We saw effects of MSR status on the linear and quadratic term.

**Term**	**Estimate**	***SE***	***t***	***p***
(Intercept)	−0.059381	0.029130	−2.038456	0.041504
mirror	−0.246222	0.078655	−3.130421	0.001746
op1	0.246440	0.058157	4.237516	0.000023
op2	−0.148520	0.041083	−3.615159	0.000300
op3	0.125062	0.034271	3.649185	0.000263
op4	0.046273	0.029130	1.588465	0.112181
mirror:op1	0.158428	0.078655	2.014229	0.043985*
mirror:op2	−0.127158	0.058157	−2.186473	0.028781*
mirror:op3	−0.050750	0.041083	−1.235310	0.216715
mirror:op4	0.047509	0.034271	1.386270	0.165665

**Figure 5.**
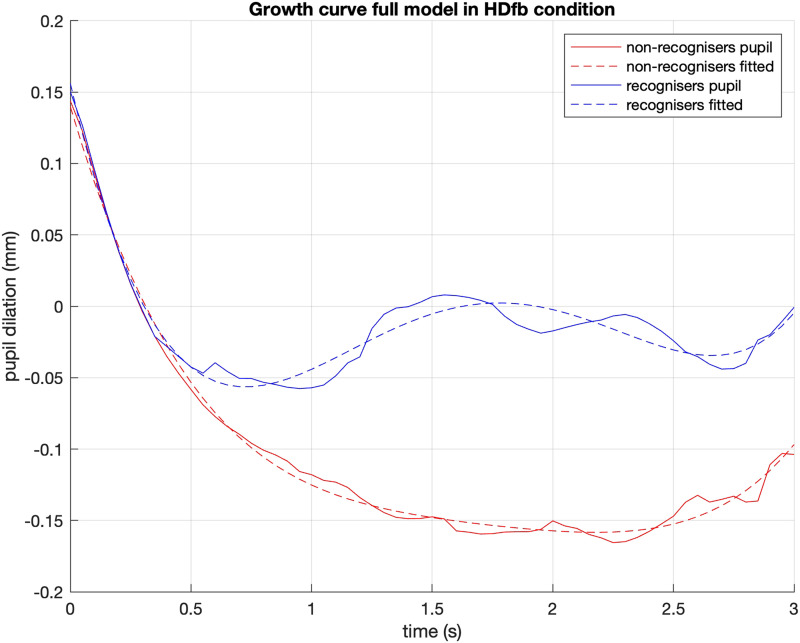
Plotted fit of the full model, between MSR groups and within the high demand condition.

We detected a significant effect of the MSR factor on the linear and quadratic term (see Table 2). This reflects the overall increase of pupil size in the recognisers compared to the non-recognisers (i.e., the effect on linear term), that is faster in the beginning (the effect on quadratic term), as we can observe in Figure 5. The difference in the pupil dynamics is indicative of the mirror recognisers, but not the non-recognisers, experiencing perspective conflict during the anticipation phase.

**Table 2.** The effect of dropping each single interaction of a polynomial term with MSR status to model fitness. Because we dropped only one term each time, the degree of freedom is one. We see that dropping the first two terms resulted in worse fitting models, which allowed us to conclude that these terms are significant.

**Interaction term dropped**	**χ^2^**	***p***
linear	3.8979	0.0483*
quadratic	4.5615	0.0327*
cubic	1.5027	0.2203
quartic	1.8850	0.1698

##### Early Effect.

**Figure 6.**
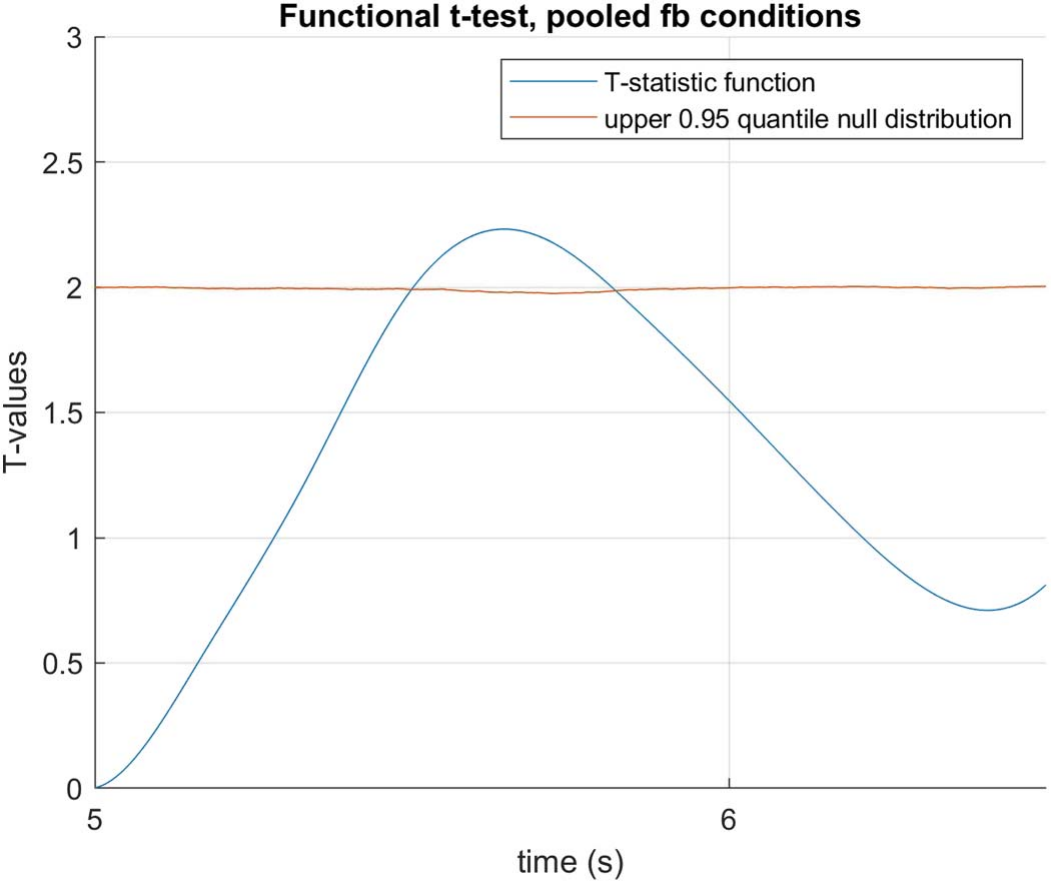
**Functional *t*-test for mirror recognisers vs. non-recognisers in both conditions in the early period around agent occlusion.** We detected a significant difference from 5498 ms to 5820 ms.

Through visual inspection of the data, we observed a difference in pupil reaction between the two mirror-status groups before our preregistered test period, at approximately the time the agent begins to be occluded (∼5–6 s). To explore this further, we conducted a functional *t*-test from 5 s to 6.5 s using permutation analysis at 0.05 significance level and 10000 permutations (see Figure 6), combining the data from both the HDfb and LDfb conditions as they are identical during this time period. We detected a statistically significant difference between the two groups from 5498 ms to 5820 ms. Taking into account the pupil responsivity delay, this suggests that the mirror recognisers showed greater pupil dilation in anticipation of the agent becoming occluded than mirror non-recognisers^2^.

### Anticipatory Looking & Inhibitory Control

We examined whether conflict between perspectives would impair children’s action prediction capabilities in the absence of sufficient inhibitory resources, predicting that infants who had achieved mirror self-recognition would show a greater tendency towards incorrect action anticipation than those who had not. We also predicted that there would be a relationship between inhibitory control and anticipatory looking on the HDfb condition, but only in those infants who had achieved self-awareness.

After pre-processing, infants in our sample watched a mean of 5.22 ± 1.93 HDfb and 5.08 ± 1.84 LDfb trials. There was no significant difference in the number of trials included per condition (*t*(98) = 0.37, *p* = 0.71). When split by MSR status, mirror non-recognisers had more trials included in both the HDfb condition (*M*_recognisers_ = 4.68 ± 1.95, *M*_non-recognisers_ = 5.76 ± 1.79, *t*(48) = 2.04, *p* = .05), and LDfb condition (*M*_recognisers_ = 4.44 ± 1.47, *M*_non-recognisers_ = 5.72 ± 1.97, *t*(48) = 2.60, *p* = .01). Both recognisers and non-recognisers anticipated in the same proportion of trials in both conditions (HDfb: *M*_recognisers_ = 0.84 ± 0.21, *M*_non-recognisers_ = 0.92 ± 0.11, *t*(36) = 1.76, *p* = .09, Welch’s test; LDfb: *M*_recognisers_ = 0.81 ± 0.20, *M*_non-recognisers_ = 0.88 ± 0.15, *t*(44) = 1.36, *p* = .18, Welch’s test).

We analysed two indicators of whether infants anticipated correctly: i) first fixation scores (i.e., whether after the cue onset, the first box they fixated on was the side where the agent should have expected to find the ball given their false belief) and ii) DLS score during the anticipation period. As our predictions differed by MSR status, we analysed each group separately.

For first fixation, recognisers were at chance in the HDfb condition and below chance in the LDfb condition (*M*_HDfb_ = 0.45 ± 0.28, *t*(24) = −0.88, *p* = .38; *M*_LDfb_ = 0.38 ± 0.24, *t*(24) = −2.60, *p* = .02; both tests are one-sample *t*-tests on first fixation scores equal to chance (.5)), and non-recognisers were below chance in the HDfb condition and at chance for the LDfb condition (*M*_HDfb_ = 0.36 ± 0.26, *t*(49) = −3.70, *p* < .001; *M*_LDfb_ = 0.41 ± 0.23, *t*(49) = −2.82, *p* = .003; both tests are one-sample *t*-tests on first fixation scores below chance (.5)).

For DLS score, recognisers were at chance in the HDfb condition and below chance in the LDfb condition (*M*_HDfb_ = −0.08 ± 0.49, *t*(24) = −0.77, *p* = .45; *M*_LDfb_ = −0.22 ± 0.47, *t*(24) = −2.40, *p* = .03; both tests are one-sample *t*-tests on DLS scores equal to chance (0), and non-recognisers were below chance in the HDfb condition and at chance in the LDfb condition (*M*_HDfb_ = −0.28 ± 0.35, *t*(24) = −4.03, *p* < .01; *M*_LDfb_ = −0.11 ± 0.28, *t*(24) = −1.99, *p* = .06; both tests are one-sample *t*-tests on DLS scores equal to chance (0). Thus, neither measure provided evidence for correct action prediction.

Given that our predictions regarding the relationship between anticipatory looking and inhibitory control assumed evidence of correct prediction with anticipatory looking, it is not informative to examine the relationship with inhibitory control abilities. We nonetheless include the preregistered analyses in the supplementary information. Of note, there were no differences in performance on the ECITT task between mirror recognisers and non-recognisers based on accuracy on prepotent trials (*t*(38) = −0.84, *p* = .41), accuracy on inhibition trials (*t*(38) = 0.32, *p =* .75), or ADS score (*t*(38) = −0.61, *p* = 0.55), indicating that inhibitory control was not related to recogniser status.

#### Exploratory Analyses

##### Latency - Anticipation Phase.

Although infants did not correctly anticipate, we considered post hoc whether examining differences in latency to make anticipatory looks would allow us to better interpret the differences in pupil dilation during the anticipation phase. Saccades during the anticipation window could reflect *either* simply a reaction to exogenous cues (e.g., the lids on the box opening or the lights on the boxes that appear at the start of this window to prompt anticipation), or *in addition*, could reflect infants’ expectation concerning in which box they think the agent will search. While neither group provided evidence that they correctly anticipated the agent’s search, we reasoned that latency to make the initial saccade could provide information on whether anticipatory saccades were exogenously or endogenously mediated. Specifically, we reasoned that a longer latency to generate a saccade to a box in the anticipatory window could reflect a delay related to managing two conflicting representations, if saccades were endogenously mediated. To examine latency, we computed the time from the box opening cue to the first saccade to one of the box AOIs. Independent samples *t*-tests (with unequal variances) revealed that mirror recognisers were indeed slower (*M* = 879.05 ± 495.64 ms) than non-recognisers (*M* = 652.97 ± 254.00 ms) in the HDfb condition (*t*(35.79) = −2.03, *p* = .05). Latency was not significantly different in the LDfb condition (*M*_recognisers_ = 810.13 ± 453.00; *M*_non-recognisers_ = 723.23 ± 250.41; *t*(37.42) = −0.84, *p* = .41).

##### Latency - Early Effect.

We also considered posthoc whether examining differences in latency to making a saccade to the agent would allow us to better interpret the differences in pupil dilation during the earlier time window, just before the agent is occluded. We reasoned that if pupil dilation reflects anticipation of the agent being occluded, we may observe differences in how quickly infants saccade to the agent. Latency was computed as the time from the curtains beginning to close to the first saccade to the agent AOI in both the HDfb and LDfb conditions combined. An independent samples *t*-test revealed that mirror recognisers were faster (*M* = 540.71 ± 270.58 ms) than non-recognisers (*M* = 712.38 ± 248.22 ms) to saccade to the agent (*t*(48) = 2.34, *p* = .02, *d* = .66).

## DISCUSSION

This study tested the hypothesis that the emergence of self-representation, between 18 and 24 months, presents a challenge for young children when they are presented with a situation in which the self and other should hold conflicting perspectives (Southgate, 2020). Under this account, self-representation enables children to represent their own perspective *as the self-perspective*, and in doing so, this representation can compete with the representation of an event that is generated from tracking the other’s perspective. Thus, we preregistered the prediction that infants who had achieved self-awareness, as indexed by mirror self-recognition, would experience greater conflict, as indexed by pupil dilation, while watching events in which conflict between self and other perspectives is high. We further predicted that this conflict would manifest in a reduced ability to make anticipatory saccades towards a location congruent with an agent’s false belief in infants who evidenced mirror self-recognition, and that this may be modulated by infants’ capacity for inhibitory control.

Our results suggest that mirror recognisers did perceive greater conflict during the late pre-registered time window (corresponding to the action anticipation phase), which reached significance only in the high demand condition in which the conflict between self and other should be particularly salient. Within this condition, mirror recognisers also took longer to saccade to a box during anticipation. While latency was not a pre-registered variable, it may provide additional support for our interpretation of the pupil dilation data. Specifically, taking longer to generate a saccade would be consistent with our interpretation that it is the mirror recognisers who detect the perspective conflict. Nevertheless, the main effect of mirror status suggests that mirror recognisers detected a conflict in both low and high demand trials. While work with adults has found that only high demand trials seem to require intact inhibitory control (Hartwright et al., 2015; Samson et al., 2005), for 18-month-olds, even the low-demand trials where they do not know the real location of the object, present a challenge. Similar to findings in adults showing that conflict manifests at the point where a response is needed (McCleery et al., 2011), the presence of a difference in pupil dilation only in this final window may imply that the perceived conflict between perspectives only emerges when the infant is prompted—albeit implicitly—to make a prediction about what the other person will likely do.

However, while we found the expected pupil dilation for recognisers in the high conflict condition, suggesting that it was only in this group and in this condition that the conflict between the two perspectives was perceived, we did not find support for our hypothesis that the non-recognisers would surpass the recognisers in correct action anticipation. To correctly anticipate, infants would ostensibly need to inhibit their own conflicting perspective. In the current study, we found either at-chance or below-chance performance on both anticipatory looking measures, depending on MSR status and trial type, suggesting that neither group predicted correctly where the agent would reach for the ball.

Considering the pupil and anticipatory looking data together, one possibility is that 18-month-olds who have achieved self-awareness begin to *perceive* a conflict but are, as yet, unable to *overcome* this conflict. However, the failure to correctly anticipate in the non-recognisers is more difficult to explain because the pupillometry data suggests that they do not perceive a conflict to the extent that recognisers do and so we predicted that they would be better able to generate correct predictions, regardless of inhibitory control capacity. We see a number of possible explanations.

First, it may be that, contrary to the altercentric bias hypothesis, infants’ failure to accurately predict is unrelated to the emergence of self-representation and what is captured here—at least on the high demand trials for non-recognisers—is evidence of a reality bias where the infant’s representation of the ball’s location is strongest from the self-perspective. However, in our exploratory analysis, we also found that non-recognisers were significantly faster than recognisers to generate a first saccade after cue onset. We ran this analysis because we reasoned that a first saccade may reflect two things: i) an attempt by the participant to predict where the agent will reach or ii) an evoked reaction to the light/sound cues and box opening, which could be random or influenced by priming of something having previously happened at one box over the other. While this analysis was exploratory and the data should be interpreted with caution, it is consistent with anticipatory looking reflecting different underlying processes in the two groups. If the recognisers perceived greater conflict, the slower latency to saccade is consistent with a genuine—if ultimately incorrect—attempt to generate a prediction. On the other hand, if the non-recognisers perceived less conflict, but still failed to correctly anticipate, it is possible that they simply default to where they know the object to be. However, given that our main prediction, that the emergence of self-representation is related to perceived conflict, was confirmed, it seems unlikely that the reason why non-recognisers perceive less conflict is because they have a clear representation of the actual location of the ball.

Interpreting the behaviour of non-recognisers as indicating that they simply fail to predict is also difficult to reconcile with data from younger infants showing that they likely do generate expectations about where someone else with a false belief will search (Onishi & Baillargeon, 2005; Southgate & Vernetti, 2014, but see non-replications such as Dörrenberg et al., 2018; Poulin-Dubois et al., 2013). Thus, while this finding requires further investigation, we believe a plausible explanation is that non-recognisers’ incorrect prediction reflects a reaction to the onset of the visual cues rather than a genuine attempt to make a prediction about where the agent will reach, and this is consistent with the faster generation of saccades in the non-recognisers. As recent studies have cast doubt on the reliability and validity of anticipatory looking as a measure of action prediction, even in simple cases where no belief evaluation is required (Kampis et al., 2021), this remains a viable interpretation.

Although we categorised infants as “recognisers” and “non-recognisers” for the purposes of this study, and our data differentiated infants on this basis, it is likely that the emergence of self-representation is gradual and protracted (de Waal, 2019; Rochat, 2003) and may be captured with other measures at an earlier time point. While self-awareness is a multi-faceted concept with different manifestations of self-awareness emerging at different time points, there is a general distinction in the literature between subjective and objective self-representation (e.g., Duval & Wicklund, 1972). For the capacity that is proposed important here, the ability to represent what one has seen as the self-perspective, it would seem likely that an objective self-representation is required. Given that mirror self-recognition is related to various behaviours that would imply an objective self-representation including the ability to see oneself in relation to others and be motivated to align with others (Kampis et al., 2022), we suggest that the ability to represent the self-perspective presupposes an objective self-representation (Southgate, 2020). It remains likely that while recognisers experienced greater conflict than non-recognisers, the non-recognisers were not devoid of self-representation entirely, and the absence of reliable belief-based action prediction in the non-recognisers may reflect this shifting balance away from an altercentric bias.

While we chose pupil dilation as a measure because of its relationship with conflict, it is important to note that we cannot be sure that pupil dilation itself reflects the process of perceiving or experiencing conflict. Pupil dilation has also been associated with both violation of expectation (Jackson & Sirois, 2009) and effort (Kaldy & Blaser, 2020) and it is unclear whether these processes are independent (Laeng et al., 2012). As we find this difference during the anticipatory time window, an alternative possibility is that it reflects the anticipatory process itself. While infants’ tendency to make anticipatory saccades did not differ between recognisers and non-recognisers nor between high and low conflict trials, it is possible that the effort involved in anticipating was greater in recognisers on the high-demand condition, precisely because this is where recognisers perceived greatest conflict. However, even if this were the case, it would still provide support for our interpretation that the emergence of self-awareness is required for infants to experience a conflict when the self and other perspectives diverge.

Finally, a finding that emerged from our exploratory analyses is that recognisers also show significantly greater pupil dilation than non-recognisers at an earlier time point which was outside of our preregistered time-windows. During this early time window, infants see the curtains begin to occlude the agent from the scene, suggesting that recognisers and non-recognisers react to the disappearance of the agent differently. While we made no a priori predictions, there are at least two possible ways of interpreting this finding. First, it may be that only the recognisers understand the significance of the disappearance of the agent, and it may be that this event is only worthy of attention if we understand the contrast with our own continued visual access. Alternatively, the difference between the recognisers and non-recognisers in pupil size during this time window could be driven by the non-recognisers’ decreased interest in events that occur in the absence of other agents. According to the altercentric hypothesis, infants encode events better when they are co-witnessed with another agent, and thus events witnessed in the absence of others receive relatively less attention (Manea et al., 2022). Whether either of these explanations has traction should be the subject of future studies.

## ACKNOWLEDGMENTS

This work was supported by a Consolidator Grant from the European Research Council (DEVOMIND 726114) to V. Southgate. The authors thank Maria Bolding Eskildsen, Kathrine Habdank, Marie Louise Krogsgård Nielsen, and Kathrine Søndergaard Christensen for their assistance with data collection, Karla Holmboe and Henrik Dvergsdal for their assistance with implementing the ECITT, and three anonymous reviewers for their helpful comments on this manuscript.

## AUTHOR CONTRIBUTIONS

E. Yeung and V. Southgate developed the study concept. All authors contributed to the study design. V. Manea and E. Yeung designed the stimuli. Data collection was performed by E. Yeung. D. Askitis and E. Yeung performed the data analysis and interpretation under the supervision of V. Southgate. E. Yeung, D. Askitis, and V. Southgate wrote the manuscript. All authors approved the final version of the manuscript for submission.

## Notes

^1^ To address the possibility that infants might infer that the agent could see the location of the ball when the boxes reopened, we asked a group of 30 adults to evaluate the stimuli. The majority of the sample did not think the agent could see inside the box (see SI for more detail).^2^ As infants saw multiple trials, they could in principle anticipate the forthcoming disappearance of the agent at the same time on each trial.
