# Supplementary material for: Emerging Self-Representation Presents a Challenge When Perspectives Conflict
Source: Open Mind (Camb). 2022 Nov 22;6:232–49. doi: 10.1162/opmi_a_00065 (PMC9692053; doi:10.1162/opmi_a_00065)
Supplement: Supplementary file 1 [file opmi-06-232-s001.docx]

**Emerging self-representation presents a challenge when perspectives conflict**

**Supplementary Information**

Emanuela Yeung, Dimitrios Askitis, Velisar Manea, and Victoria Southgate

Department of Psychology, University of Copenhagen

**Method**

**Participants**

To reach our pre-registered sample of 50 infants (n = 25 in each group of mirror recognisers and mirror non-recognisers) we tested 80 infants in total. Participants were included based on providing sufficient eye-tracking data for analysis (described below) and completing the Mirror Self-Recognition (MSR) task. Of the 30 excluded participants, n = 29 did not provide enough eye-tracking data, n = 1 participant provided enough eye-tracking data and completed the MSR task but was not included as we had already reached our pre-registered sample of 25 mirror non-recognisers.

**Non-Verbal Perpsective-Tracking Task**

*Exclusion criteria*

Infants were excluded if they looked away for more than 1.5s (consecutively) during the portions of the test trials that were critical for tracking the location of the ball. In both conditions these were seconds 3.0-5.0 for the first movement and 6.3-9.0 for the second movement. In either of the two familiarization trials, infants were excluded if they looked at the screen for less than 2s (cumulative) during the portion of the trials sufficient to track the location of the ball and the box that agent was reaching into (seconds 3.0-6.8).

*Pupil data pre-processing*

The raw pupil data was converted from arbitrary units to millimeters using the following formula that we derived using artificial pupils:

$$Millimeters = 0.14211104 + 1.0689171 \times\frac{AU}{1000}$$

Next, we examined the data obtained from each eye for each participant and excluded data from one eye when the ratio of the pupil standard deviations between eyes was less than 1.5, considering that variability of each eye is usually similar to the other, and that large difference in variability seemed to reflect very poor quality data from one eye.

The subsequent steps of our pre-processing pipeline followed the procedure described by Kret and Sjak-Shie (2018): first, we used the following steps to identify and remove outliers: 1) pupil data smaller than 1.5mm and larger than 9mm were removed, 2) a dilation speed maximum threshold of 16 median absolute deviations was applied, 3) a smooth trend-line deviation filter was repeated 4 times to further exclude deviating data that was clustered together, and 4) temporally-isolated samples (larger than 40ms in distance, and less than 50ms in width) were removed. Gaps in the resulting data under 400ms were interpolated, and a mean pupil signal was computed from the data of the two eyes. The resulting data were smoothed using a 4Hz low pass filter (Jackson & Sirois, 2009).

*Gaze data AOIs*

We defined an AOI that included the two boxes. Box AOIs were equal-sized squares centered on each box with an additional 36-pixel wide margin in each direction

**Early Childhood Inhibition Touch Screen Task**

*Materials and Procedure*

The task was presented on an 11” Apple iPad tablet, which the experimenter held in front of the child, who was seated on their parent’s lap. In this task, children see two buttons on the screen and are instructed to “press the happy face”. The task began with 4 practice trials in which a single blue “smiley” icon was presented on the centre of the screen. On the first practice trial, the experimenter demonstrated that tapping this icon would result in a short cartoon animation with sound effects. On the second trial the child was encouraged to “press the happy face”. If the child was reluctant to press the button, the experimenter demonstrated again (for up to 3 more trials) until the child was happy to press the button on their own.

After the 4 practice trials, a single block of 32 experimental trials were presented. On each trial two buttons were shown on the screen and the child was instructed to “press the happy face”. If the child tapped the correct button, an animation played, after which the next trial began immediately. If the child tapped the incorrect button, the buttons disappeared from the screen for 1s before the next trial began. The smiley button appeared in the prepotent location on 75% of trials (24 trials) and in the inhibitory location on 25% of trials (8 trials). The experimental blocks always began with at least 3 prepotent trials. On the first trial the experimenter pointed to the correct response location to ensure that the child responded to the prepotent location from the beginning; this first trial was always removed from the analysis. To ensure a high level of participant engagement, the experimenter reminded the child to “press the happy face” as needed throughout the testing session and provided encouraging comments when the reward animations were shown.

Performance was measured based on accuracy, with a higher score indicating lower inhibitory control^[[1]](#footnote-1)^. Accuracy was measured by computing an accuracy difference score (ADS) based on subtracting the percentage correct on inhibitory trials from the percentage correct on the prepotent trials. Trials with reaction times shorter than 300ms were excluded following Holmboe et al. (2021). Participants were also excluded if they did not understand or cooperate with the task instructions, based on the criterion of more than 60% correct on prepotent trials.

**Results**

**Pupil Dilation Across Three Pre-Registered Time Windows**

**ANOVA Tables**

Table 1. Repeated-Measures ANOVA for Window 1

| **Within Subjects Effects** | | | | | | | | | | | | | |
| --- | --- | --- | --- | --- | --- | --- | --- | --- | --- | --- | --- | --- | --- |
| **Source** | **Sum of Squares** | | | **df** | | **Mean Square** | | **F** | | **p** | | **η²** | |
| condition |  | 0.006 |  | 1 |  | 0.006 |  | 1.255 |  | 0.268 |  | 0.003 |  |
| condition ✻ mirror |  | 0.0001 |  | 1 |  | 0.0001 |  | 0.030 |  | 0.863 |  | 0.00008 |  |
| Error |  | 0.236 |  | 48 |  | 0.005 |  |  |  |  |  |  |  |
|  | | | | | | | | | | | | | |
| *Note.*  Type III Sum of Squares | | | | | | | | | | | | | |

| **Between Subjects Effects** | | | | | | | | | | | | | |
| --- | --- | --- | --- | --- | --- | --- | --- | --- | --- | --- | --- | --- | --- |
| **Source** | | **Sum of Squares** | | **df** | | **Mean Square** | | **F** | | **p** | | **η²** | |
| mirror |  | 0.002 |  | 1 |  | 0.002 |  | 0.053 |  | 0.819 |  | 0.001 |  |
| Error |  | 1.614 |  | 48 |  | 0.034 |  |  |  |  |  |  |  |
|  | | | | | | | | | | | | | |
| *Note.*  Type III Sum of Squares | | | | | | | | | | | | | |

Table 2. Repeated Measures ANOVA for Window 2

| **Within Subjects Effects** | | | | | | | | | | | | | |
| --- | --- | --- | --- | --- | --- | --- | --- | --- | --- | --- | --- | --- | --- |
| **Source** | | **Sum of Squares** | | **df** | | **Mean Square** | | **F** | | **p** | | **η²** | |
| condition |  | 0.008 |  | 1 |  | 0.008 |  | 0.890 |  | 0.350 |  | 0.004 |  |
| condition ✻ mirror |  | 0.0008 |  | 1 |  | 0.0008 |  | 0.088 |  | 0.768 |  | 0.0004 |  |
| Error |  | 0.425 |  | 48 |  | 0.009 |  |  |  |  |  |  |  |
|  | | | | | | | | | | | | | |
| *Note.*  Type III Sum of Squares | | | | | | | | | | | | | |

| **Between Subjects Effects** | | | | | | | | | | | | | |
| --- | --- | --- | --- | --- | --- | --- | --- | --- | --- | --- | --- | --- | --- |
| **Source** | | **Sum of Squares** | | **df** | | **Mean Square** | | **F** | | **p** | | **η²** | |
| mirror |  | 0.006 |  | 1 |  | 0.006 |  | 0.210 |  | 0.649 |  | 0.003 |  |
| Error |  | 1.479 |  | 48 |  | 0.031 |  |  |  |  |  |  |  |
|  | | | | | | | | | | | | | |
| *Note.*  Type III Sum of Squares | | | | | | | | | | | | | |

Table 3. Repeated Measures ANOVA for Window 3

| **Within Subjects Effects** | | | | | | | | | | | | | |
| --- | --- | --- | --- | --- | --- | --- | --- | --- | --- | --- | --- | --- | --- |
| **Source** | | **Sum of Squares** | | **df** | | **Mean Square** | | **F** | | **p** | | **η²** | |
| condition |  | 0.001 |  | 1 |  | 0.001 |  | 0.079 |  | 0.780 |  | 0.0004 |  |
| condition ✻ mirror |  | 0.016 |  | 1 |  | 0.016 |  | 0.863 |  | 0.358 |  | 0.004 |  |
| Error |  | 0.892 |  | 48 |  | 0.019 |  |  |  |  |  |  |  |
|  | | | | | | | | | | | | | |
| *Note.*  Type III Sum of Squares | | | | | | | | | | | | | |

| **Between Subjects Effects** | | | | | | | | | | | | | |
| --- | --- | --- | --- | --- | --- | --- | --- | --- | --- | --- | --- | --- | --- |
| **Source** | | **Sum of Squares** | | **df** | | **Mean Square** | | **F** | | **p** | | **η²** | |
| mirror |  | 0.101 |  | 1 |  | 0.101 |  | 1.652 |  | 0.205 |  | 0.026 |  |
| Error |  | 2.926 |  | 48 |  | 0.061 |  |  |  |  |  |  |  |
|  | | | | | | | | | | | | | |
| *Note.*  Type III Sum of Squares | | | | | | | | | | | | | |

*Growth Curve Analysis*

We used normalised orthogonal polynomial terms up to degree 4 to model the pupil data from 14.5s to 17.5s. Degree 4 was decided by comparing model fits of the combined data from both demand conditions while adding higher polynomial terms to the null model repeatedly, in which model fit improved significantly only until adding the fourth degree polynomial.

The different degree polynomials can be interpreted in the following way: 1) the first degree is intended to model the slope of the linear trend of the data, 2) the second degree reflects the degree of steepness of the change in the data (meaning that smaller magnitude reflects a flatter curve), and 3) higher degrees reflect the existence of inflection points in the data time series (see also Mirman, 2016).

To model our data, we first fit models based on our 2x2 mixed effects design, considering all mirror groups, demand conditions, and their interaction, which did not give any significant result. Considering our study may be underpowered, and that the LDfb condition failed to show any significant effects in the pre-registered time windows on its own, we decided to examine the HDfb condition separately.

Within the HDfb condition, we considered the full model with mirror status as a main effect and all its interactions with polynomial terms (see Table 1). Based on a Likelihood Ratio Test, the full model fit the data better than the null model (i.e., the one consisting of the polynomial predictors but without the mirror factor; 𝛘² = 11.416, dDF = 5, p <0.05). Following Mirman (2017), we examined each individual interaction by dropping it from the full model, and evaluated its unique effect by the reduction in the model fit, which determined statistical significance for the interaction of the group factor on the linear and quadratic term (see Table 2). These reflect some characteristics of the shape of the pupil time course in the outcome time window (see Figure 5), i.e. the effect on the linear term reflects that the non-recognisers’ pupil course tends to show larger overall decrease than the recognisers’ course, while the effect on the quadratic term reflects that the pupil course is flatter in the recognisers and steeper (i.e. more “U” shaped) in the non-recognisers.

**Anticipatory looking scores between groups**

When we compared anticipatory looking scores between groups, an independent samples t-test revealed that mirror recognisers had significantly higher first fixation scores in the HDfb condition compared to mirror non-recognisers (*M_r_*_ecognisers_ = -0.08 ± 0.46; *M*_non-recognisers_ = -0.28 ± 0.35; t(48) = -2.48, p = .02, d = -0.70).

**Relationship between inhibitory control and anticipatory looking**

After exclusion, we had inhibitory control data from 40 infants (n = 18 non-recognisers and n = 22 recognisers). Overall infants were more accurate on prepotent trials (proportion correct = 0.86 ± 0.13) compared to inhibition trials (proportion correct = 0.57 ± 0.35), and there were no differences in performance between mirror recognisers and non-recognisers based on accuracy on prepotent trials (t(38) = -0.84, *p* = .41), accuracy on inhibition trials (t(38) = 0.32, *p =* .75), or ADS score (t(38) = -0.61, *p* = 0.55).

When we examined the relationship between inhibitory control and anticipatory looking, there was a positive correlation between ADS and DLS in the LDfb condition (r = .32, p = .05). This seemed to be driven by the correlation between ADS and DLS in mirror recognisers in the LDfb condition (r = .45, p = .04) as there were no significant correlations between any measure of anticipatory looking and inhibition in mirror non-recognisers. This positive correlation suggests that mirror recognisers with poorer inhibitory control skills were more likely to look first to the box that was congruent with the agent’s false belief.

**Discussion**

We asked 30 adults (27 female, 3 male, *M*_age_ = 34 ± 1 years), who were naive to the purpose of the study, to evaluate the stimuli videos. Half the group saw the high demand video, and half saw the low demand video. In total 22/30 (73%) adults did not think that the agent could see inside the box.

**References**

Holmboe, K., Larkman, C., de Klerk, C., Simpson, A., Bell, M. A., Patton, L., Christodoulou, C., & Dvergsdal, H. (2021). The early childhood inhibitory touchscreen task: A new measure of response inhibition in toddlerhood and across the lifespan. *PLoS One*, *16*(12), e0260695. https://doi.org/10.1371/journal.pone.0260695

Jackson, I., & Sirois, S. (2009). Infant cognition: going full factorial with pupil dilation. *Developmental Science*, *12*(4), 670-679.

Kret, M. E., & Sjak-Shie, E. E. (2019). Preprocessing pupil size data: Guidelines and code. *Behavior research methods*, *51*(3), 1336-1342.

Mirman, D. (2017). *Growth curve analysis and visualization using R*. Chapman and Hall/CRC.

1. We preregistered reaction time as an additional measure of performance. However, we decided not to include this measure as Holmboe et al. (2021) found that reaction time is not a reliable measure in children under the age of 2 years. [↑](#footnote-ref-1)
